# Elucidating the functions of gut microbiota from two edible dung beetle species: Implications for waste management and food industry

**DOI:** 10.1371/journal.pone.0325756

**Published:** 2025-06-25

**Authors:** Syombua S. Mwanza, Cynthia M. Mudalungu, JohnMark Makwatta, James Kabii, Njogu M. Kimani, Chrysantus M. Tanga

**Affiliations:** 1 International Centre of Insect Physiology and Ecology (*icipe*), Nairobi, Kenya; 2 Department of Physical sciences, University of Embu, Embu, Kenya; 3 Department of Chemistry and Material science, Technical University of Kenya, Nairobi, Kenya; Zhejiang A and F University, CHINA

## Abstract

Dung beetle larvae are consumed globally, yet their gut microbiota across different geographical locations remains largely unexplored. This study examined the diversity and composition of the gut microbiota of two edible dung beetle species, *Cetonia aurata* and Rhinoceros beetle (*Oryctes* sp.), from three sites in Kenya. Using advanced molecular techniques, bacterial and fungal communities were sequenced. The most abundant bacterial phyla in *C. aurata* were Firmicutes (42.10%) and Bacteroidota (32.50%), while *Oryctes* sp. had higher levels of Proteobacteria (35.00%), Actinobacteriota (11.40%) and Desulfobacterota (7.40%). Fungal communities were dominated by Lecanoromycetes (92.60%) in *Oryctes* sp. and Saccharomycetes (92.60%) in *C. aurata*. Beta diversity analysis revealed that neither species phylogeny nor larval location significantly influenced the presence of specific microbes. Pathways related to nitrogen and carbon degradation were predicted in bacteria like *Bacillus*, *Pseudomonas mosselii*, and *Proteiniphilum*. This implies that bacteria and fungi from the gut of dung beetle larvae could be ideal targets for potential bio‐resource to eliminate waste pollution and opened an important window of opportunity for bio-based economy solutions. This work offers very practical solutions that have great business potential that can create a gigantic market ranging from functional foods to dietary supplements and therapeutic applications.

## Introduction

The gut consortia in insects comprise of microbes including protozoa, bacteria, fungi and archaea [1]. These microbial communities play crucial roles in insect functioning that can be exploited for various perspectives including ecology, agriculture and medicine. In medicine, insect symbionts influence vectoring efficiency and parasite developmental time, hence providing potential targets for disease control [2]. Bio-geochemical cycles such as carbon cycling, nitrogen cycle and plant biomass degradation also critically depend on insects and their microbial communities [3].

The acquisition of gut microbes is influenced by a variety of factors, such as nutritional requirements, behavior and living environments. Furthermore, the composition, abundance, and stability of gut microbial communities are shaped by various endogenous and exogenous factors, including environment, diet, and host phylogeny [4]. For instance, the passalid beetle *Odontotaenius disjunctus* requires *Lactococcus* and *Turicibacter* bacterial microbes to break down wood fibers [5]. Similarly, termites that are incapable of breaking down lignocellulose develop intestinal microbes to perform this vital task thus enabling them to consume wood [6].

Diverse gut bacteria found in different termites and cockroaches demonstrated that host phylogeny has a significant role in determining the composition of the gut communities [7]. Moreover, the gut bacterial compositions of two African con-generic dung beetle species (*Pachysoma*) and the four fruit borers were found to be significantly influenced by diet [8,9]. The plasticity of gut microbiota often due to diet changes enables insects to exploit variety of food sources. This introduces the basis of host differentiation, providing an ecological rationale behind the high diversity among insects.

Coleoptera is one of the most successful insect orders, consisting of more than 370,000 species globally identified. These species are saprophagous and free-living in soil, their main food being either wet or dried fecal matter from herbivorous mammals [10]. They utilize mammalian excreta that remains undigested as a source of nourishment and habitat across their life-cycle thereby underscoring their ecological significance. Beetles play a vital role in cellulose biodegradation, increasing bioturbation and suppressing gut parasites in their environs [11]. Their involvement in the breakdown of lignocellulosic material presents a sustainable and environmentally friendly approach to waste management, potentially attributable to microbial fermentation, given their lack of active endogenous cellulase enzymes [12]. Additionally, beetle larvae possess an incarnate defense system to enable them to live in hostile environments as well as being in myriad relationships with gut microbes [13]. These adverse environs push the organisms to evolve since they face challenges in responding to the environmental influences which affect host development.

Despite the increased interest and numerous studies in insect gut microbial composition [13], the ecological functions offered by dung beetles and the biodiversity of their gut microbiota remain largely understudied. Moreover, researchers have focused on culturable plant cell wall degrading microbes associated with root-feeding pests, such as scarab beetles or the transfer of gut microbes from female adults to larvae in beetle species [14–16] leaving a paucity of information about variations in their gut microbial communities and the lignin/nitrogen-degrading metabolic pathways associated with them.

We hypothesized that beetle larvae from geographically isolated (allopatric) regions would exhibit greater differences in gut microbial composition; however, if host phylogeny primarily shapes gut microbiota, then two saprophagous species should have similar microbial communities regardless of their region of origin. Herein, a complete metagenomics of the gut DNA extracts from two coleopteran beetle larvae was investigated. The study details the characterization of both eukaryotic and prokaryotic communities using a combination of molecular techniques, along with the prediction of their functional roles. The findings considerably improve our understanding of variations in the microbiome of Scarabaeoid beetle larvae, while also providing unique perspectives and highlighting their significance to the field of biotechnology.

## Materials and methods

### Sample collection

**Fig 1:**
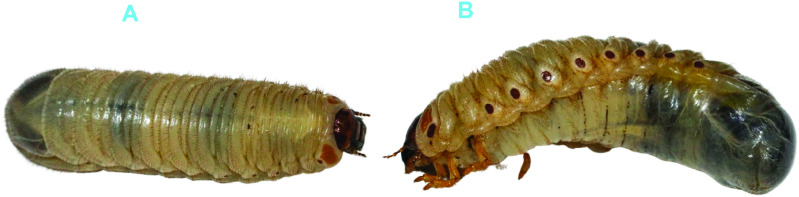
Photographs of morphologically distinct scarab beetle larvae, namely *C. aurata* (A) and *Oryctes* sp. (B). Note. In the third instar, *C. aurata* larvae measure 55 mm in length, have a diameter of 35 mm, and possess three pairs of limbs. In contrast, *Oryctes* sp. larvae measure 85 mm in length, have a diameter of 45 mm, and exhibit three pairs of limbs.

The late second to third instar morphologically distinct coprophagous scarab beetle larvae (Fig 1) were collected across three counties between January and February 2023. The sampling regions included Embu County [Nthangaiya (S00˚27’58.9”, E037˚33’58.6”), University of Embu (S00˚30’41.3”, E037˚27’29.5”) and Gachururiri (S00˚42’25.6”, E037˚28’58.7”)]; Murang’a County [Kiunyu (S00˚57’21.8”, E037˚1’9.2”), Njoguini (S00˚43’17.7”, E037˚7’38.2”) and Kairi (S00˚36’48.3”, E037˚0’44.3”)] and Nairobi County [Mwiki (S01˚13’47.2”, E036˚57’1.0”), Ruai (S01˚17’27.7”, E037˚0’29.6”) and Kangemi (S01˚15’53.9”, E036˚44’37.5”)]. In each county, there were three sites (n = 10 larvae per site), though in some sites, *C. aurata* larvae were absent. These larvae gut microbiome were compared with the larvae collected at the other six allopatric sites in the aforementioned counties. Beetle larvae species were collected from dung produced by livestock grazing on pastures and vegetation found in each county. These pastures were subjected to a range of climatic conditions, including savanna in Embu, equatorial in Murang’a, and subtropical highland in Nairobi. However, in Nairobi, the substrates in which the larvae were obtained from consisted of fruit peels, food remnants and livestock dung.

### Sample preparation and pre-processing

The captured larvae were housed in plastic containers and provided with their fresh organic material (cattle dung and vegetable waste) until processing to minimize differences in gut microbiota composition as a result of diet changes until dissection [8]. Larvae dissection was done according to the protocol described by [17]. The entire guts of five larvae per site were dissected and collected from all live individual samples within 24 hours of capture and starvation. Dissections were performed in sterile glassware using micro-scissors and forceps that were surface sterilized with 40% bleach and 70% ethanol, ensuring that all procedures were conducted with thoroughly cleaned tools. The larvae were briefly anesthetized for 15 minutes in −20 °C freezer and upon removal, they were thoroughly rinsed with tap water before placing them on sterile preparation dishes. They were then surface-sterilized with 70% ethanol for 50 seconds and then rinsed in six successive changes of sterile distilled water. The cuticle was then sliced along the lines and the ventral integument allowing the ring-shaped muscles to be removed. The head was removed and a circular cut performed on the anus to allow the intestinal gut to be extracted. The whole guts from *C. aurata* (45−50 mm long) and *Oryctes* sp. (60−65 mm long) were stored in sterile 15 mL falcon tubes at −20 °C until DNA extraction. The two beetle larvae employed in this study were previously identified as *Cetonia aurata* (A) and *Oryctes* sp. (B). Their sanger sequences can be obtained under the accession numbers OQ925397.1 and OR115609.1, respectively.

### Genomic DNA extraction from gut samples

DNA was extracted from each sample using CTAB – phenol- chloroform method as outlined in [18]. First, individual thawed gut samples were homogenized using Qiagen TissueLyser II. To the 250 µL of the homogenate from each sample, an equivalent quantity of pre-chilled CTAB buffer was added. The mixture was then filled up with warmed CTAB buffer and digestion enzyme (25 µL of proteinase K) then incubated at 65 °C for 15 minutes with continuous shaking after every 5 minutes. The supernatant was centrifuged at 1500 rpm and then transferred to a clean 2 mL Eppendorf micro-centrifuge tube. One volume chloroform – isoamyl alcohol (24:1) was added to the supernatant followed by inversion of the tubes severally to minimize protein contamination. This was followed by centrifugation at 1500 rpm after which the supernatant was transferred to a sterile1.5 mL Eppendorf micro-centrifuges tubes. To precipitate the DNA, 70% isopropanol (v/v) was added to the supernatant and inverted several times. The mixture was centrifuged at 1500 rpm for 8 minutes to create a pellet, and the supernatant carefully discarded. The pellet was rinsed twice with 250 mL of 70% ethanol followed by one wash with 100% ice cold ethanol and centrifugation steps of 1500 rpm per wash. After air-drying, the pellets were dissolved in EB elution buffer (Meridian Bioscience). The phenolic compounds were removed from DNA using agarose gel electrophoresis (1%) modified with 1% polyvinylpyrrolidone (PVP). The addition of PVP helps in retarding the electrophoretic mobility of humic and fulvic acids, preventing comigration with the high molecular weight DNA [19]. The DNA was recovered from the gels and quantified using NanoDropTM 2000 UV-Vis spectrophotometer (Thermo Fischer Scientific, Wilmington, USA). DNA samples with good quality ranging from 1.7–2.1, based on A260/A280 nm, were selected for 16S rRNA (bacteria) and 18S (eukaryotes) and stored at −80 °C until processing. Afterward, approximately 80 µL of the DNA obtained was sent to Macrogen Inc (Netherlands) for Illumina next-generation sequencing (NGS).

### Library preparation, PCR and sequencing of the bacterial 16S rRNA gene and eukaryotic 18S rRNA region

Amplicon sequencing was carried out on the Illumina Miseq platform targeting 16s and V4R regions according to the manufacturer’s protocol. For 16S library preparation, the primer pair 341F (CCTACGGGNGGCWGCAG) and 805R (GACTACHVGGGTATCTAATCC) [20] primers were used to target the V3–V4 region. The primer pair V4F (CCAGCASCYGCGGTAATTCC) and V4R (ACTTTCGTTCTTGATYRA) which flank the 18S ribosomal RNA (18S rRNA) was utilized for the preparation of the 18S library [21]. The sequencing process involved paired-end two 300–cycle sequencing, utilizing a 600–cycle v3 sequencing kit.

### Bioinformatics

FASTQC (v.0.11.6) was used to assess the quality of raw sequence reads (Wingett & Andrews, 2018). Pre-processing of the raw reads was carried out using the Divisive Amplicon Denoising Algorithm (DADA2) (v 1.28.0). Workflow proposed by [22] was used to process raw reads in R v4.3.0 using the R Studio v2023.06.0 interface (R Core Team, 2023). Cutadapt (v4.6) was used to trim, dereplicate and perform error rate reading at 0.2 from our raw sequences [23]. Trimming and filtering out of 16S and 18S sequence reads were performed with the following custom parameters: forward reads at 250 base pairs, reverse at 160 base pairs, maxN = 0, maxEE= (2,5) and truncQ = 2. Low quality reads were then eliminated to increase the merging chances and the accuracy of error learning in DADA2. Demultiplexing was carried out using the “derepFastq” function then the ‘RemoveBimeraDenovo’ function employed to eliminate spurilous and chimeric reads to ensure only high-quality sequence reads were inferred into their associated amplicon sequence variants (ASVs). Phylogeny was assigned against pretrained databases; SILVA (v 138.2) (16s) [24] and Protist Ribosomal Reference (18s) [25] based on pairwise identification using the ‘assign Taxonomy’ function. Multiple sequence alignment was done using the ‘AlignSeqs’ function in DECIPHER package v2.28.0 [26]. Further analyses, manipulation and data visualization were performed using phyloseq v1.44.0 [27], Tidyverse package v2.0.0 [28], metagMisc v0.5.0 [29] and Janitor package v2.2.0 [30].

Two phyloseq objects for both bacteria and protists were created followed by eliminating all ASVs corresponding to undesired sequences such as chloroplast, mitochondria and archaea using the “subset_taxa” function of phyloseq. Subsequent taxa were then further filtered to ensure that only all the most abundant taxa were retained. The metadata, ASVs and taxonomy tables were combined to create a phyloseq [27] object to aid in visualization. To visualize the structure of the gut microbial communities, a stacked bar plot was created using the thirty most abundant reads. The mean ASV abundance for each sample was obtained by rarefying to a sampling depth of 6500 reads using ‘rarefy_even_depth’ function and ‘vegan’ package [31] to minimize the effects of uneven sequence counts between the samples. Stacked bar plots were used to show the relative abundance of bacterial communities at genus level. Alpha diversity was determined using the Microbiota process v1.9.3 based on sample ASV profiles from a rarefied phyloseq object using; “Chao1,” “abundance-based coverage estimator (ACE),” “Pielou’s Evenness,” “Simpson Evenness” and “Shannon” diversity predictors. The resultant indices were then subjected to Shapiro-Wilk test to assess normality before visualization using the “ggpubr” package v0.6.0 [32]. Effects of beetle larvae genus and geographical location on the alpha diversity were then assessed using Kruskal-wallis pairwise comparisons. Beta-diversity (β-diversity) was computed using the Weighted UniFrac distance, to assess the clustering pattern of bacterial communities using the using the “phyloseq::ordinate” function. This was visualized using principal-coordinate analysis (PCoA) biplots using the ‘vegan’ package v2.6.4 in R [31]. Defining core microbiota was done by making detailed comparisons of bacterial species identified at the genus level to describe the number of shared ASVs. For this, Venn diagrams were generated using the ‘Venn Diagram’ package v1.7.3 [33] to illustrate the core shared bacterial genera, which were classified as those that had a 30% prevalence in both larvae species. Permutational multivariate analysis of variance (PERMANOVA) with Betadisper was used to detect variations within the gut microbiota between the larvae sampled from different locations using the “Adonis” function of “vegan” package (v2.6.4) package in R [31].

### Functional prediction of the detritivores bacterial gut microbiota

The phylogenetic Investigation of Communities by Reconstruction of Unobserved counts per sample (PCRUSt2, version 2, [34]) was used to predict the functional analysis of the bacterial gut microbiota. The predicted gene family-counts per sample were tabulated using DC reads abundance, orthologous groups and identifiers constructed with Kyoto Encyclopedia of Genes and Genomes (KEGG). The categories unrelated to bacterial metabolism and physiology were removed after level 3 classification of KEGG identifiers. Results from level 3 categories were visualized by generating a heatmap using STatistical Analysis of Metagenomic Profiles (STAMP) software [35]. Box plots in STAMP were utilized to identify pathways with significant differences between the two saprophagous larvae.

### Ethics statement

Institutional Review Board Statement: The Authority to conduct the experiment and collect data was in accordance with the animal welfare regulations and granted by National Commission for Science, Technology, and Innovation (NACOSTI); Research Permit License No: NACOSTI/P/23/27477. This research also received approval from the Institutional Animal Care and Use Committee (IACUC) of Kenya Agricultural and Livestock Research Organization (KALRO)-Veterinary Science Research Institute (VSRI); Muguga North upon compliance with all provisions vetted under and coded: KALRO-VSRI/IACUC028/16032022.

## Results

### Classification of gut microbial communities of the scarab beetles

Two coleopteran larvae, *C. aurata* and *Oryctes* sp. share similar lifestyle, by feeding on either fresh or decomposing animal waste in the soil. They also live in similar ecological niche despite of occurring in different counties in Kenya. Their gut microbiomes obtained from different geographical sites were determined using 16S/18S rRNA gene amplicon sequencing. The number of bacterial sequences considerably varied between different samples, ranging from 6044 to 18978 for *C. aurata* and 10170–17976 for *Oryctes* sp. (Table 1). In terms of fungal species, *Oryctes* sp. sequences ranged from 151 to 27564, while *C. aurata* sequences spanned from 3577 to 100236 (S1 Table in S1 File). The ACE (Table 1) and rarefaction curves (S1A Fig in S1 File) indicated that the bacterial gut communities were adequate for a reliable comparison of the bacterial gut communities.

**Table 1:** Total number of rarified bacterial sequences and species richness of bacterial communities in both Scarabaeoid beetle larvae (n = 5), sequenced as a pool.

Species	Collection locality	Total no. of rarefied bacterial sequences	ACE	Chao1	Shannon	Pielou
*Oryctes* sp.	Murang’a	10170	300.50	300.75	5.48	0.96
*Oryctes* sp.	Embu	14766	391.06	392.53	5.55	0.93
*Oryctes* sp.	Murang’a	15169	218.59	222.24	4.38	0.82
*Oryctes* sp.	Embu	17976	369.41	369.62	5.67	0.96
*Oryctes* sp.	Embu	17162	502.59	504.15	5.69	0.92
*Oryctes* sp.	Murang’a	25404	400.00	400.00	5.75	0.96
*Oryctes* sp.	Nairobi	12720	418.57	418.55	5.77	0.96
*Cetonia aurata*	Embu	10656	305.46	305.60	5.41	0.95
*C. aurata*	Murang’a	10690	588.30	594.22	5.62	0.89
*C. aurata*	Embu	6044	302.70	303.20	5.52	0.97
*C. aurata*	Murang’a	30205	252.00	252.00	5.29	0.96
*C. aurata*	Nairobi	18978	436.73	440.53	5.51	0.91
Geographical site effect		*χ*²		7.87	2.90	7.10
		Pr(<or >F)		0.04	0.08	0.03
		Df		2	2	2
Host phylogeny		*χ*²		0.33	3.30	0.56
		Pr > F		0.56	0.07	0.45
		Df		1	1	1

**Note.** The total count of rarefied sequences identified in each species is provided. The ACE and Chao1 values serve as indicators of species richness. Kruskal-Wallis tests revealed significant differences in species evenness across the board. Evenness values ranging from 0 to 1 suggest that the bacterial communities are distributed evenly.

**Fig 2:**
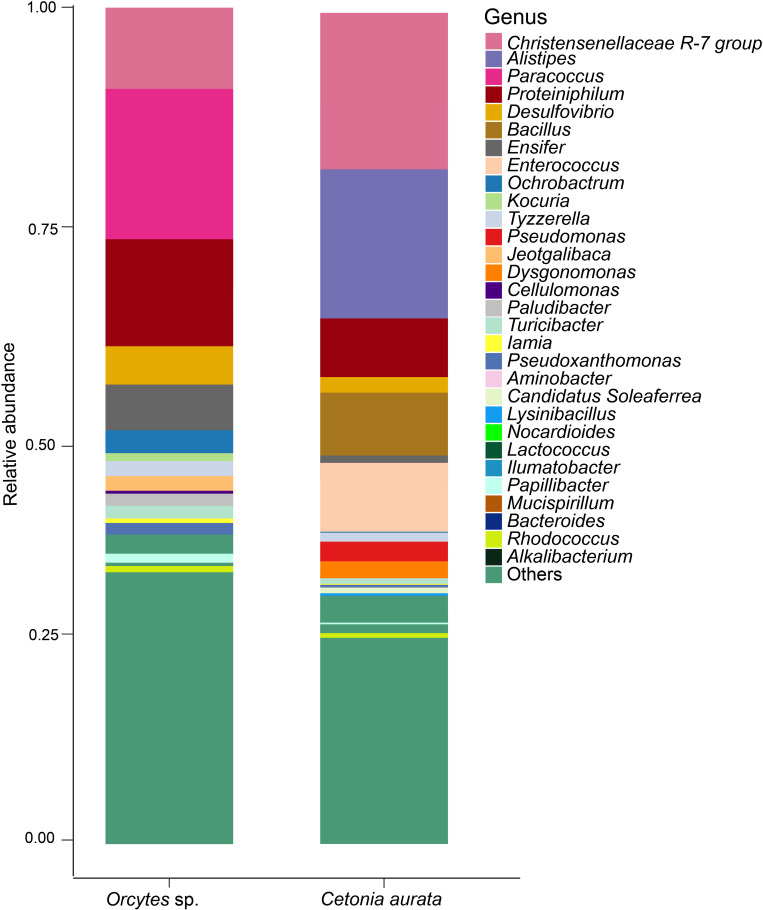
Taxonomic composition of bacterial communities associated with Scarab beetle larvae. **Note.** Bar plots representing the relative abundance of the predominant bacterial amplicon sequence variants at the genus level between the *Oryctes* sp. and *C. aurata* larvae. “Others” refers to all ASVs with an initial relative abundance below 1%.

Across the samples collected from the different sites, about 3871 genera level taxa were identified, representing 5 taxa of Archean and 3866 taxa of bacteria. The larvae from *C. aurata* and *Oryctes* sp. were dominated by similar bacterial phyla and genera, however, the relative abundances of the communities differed between the beetle species. Among the 14 phyla found, Firmicutes (42.10%) and Bacteroidota (32.50%) were the most prevalent in *C. aurata*, whereas Proteobacteria (35.00%), Actinobacteriota (11.40%), and Desulfobacterota (7.40%) were prominent in *Oryctes* sp. (S2 Table in S1 File). There were 23 classes found in the ASVs, with Alphaproteobacteria (29.60%) and Actinobacteria (8.60%) being the most common in *Oryctes* sp. compared to *C. aurata*, which had Bacteroidia (32.50%), Bacilli (22.40%), and Clostridia (19.20%) (S3 Table in S1 File). In terms of genera, *Paracoccus* > *Proteiniphilum* > *Christensenellaceae* R-7 group > *Desulfovibrio* > *Ensifer* had the highest relative abundances in *O. rhinoceros*. On the other hand, *C. aurata* had *Alistipes* > *Christensenellaceae* R-7 group> and *Bacillus* > *Enterococcus* as the most abundant groups (Fig 2, S4 Table in S1 File).

**Fig 3:**
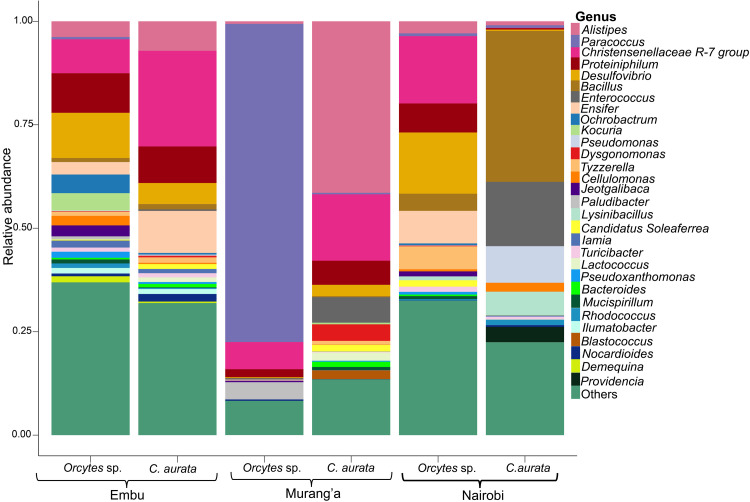
Stacked bar plots displaying the taxonomic profiles of the thirty most abundant bacterial communities associated with the guts of *Oryctes* sp. and *C. aurata* collected from the same region. **Note.** Bacteria that could not be identified at the genus level were grouped under ‘Others.

Besides the previously listed species, the presence of distinct bacterial communities was noted in sites like Nairobi that were more prevalent than the rest of the areas: *Bacillus* (36.5%) in *C. aurata*, *Turicibacter* (1.3%) and *Desulfovibrio* species (14.8%) in *Oryctes* sp. *Dysgonomonas* (4.0%), *Candidatus Saccharimonas* (1.6%), and *Blastococcus* (2.10%) were the most prevalent in Murang’a *C. aurata* larvae. In Embu, it was *Nocardioides* (1.8%), *Ensifer* (10.2%) from *C. aurata* and *Iamia* (1.7%) from *Oryctes* sp. (Fig 3). For these allopatric sites (Fig 3) relative abundances drove the main difference rather than presence or absence of specific microbes.

According to the scatter dot plot (S2 Fig and S5 Table in S1 File), the most common bacterial species in *C. aurata* were *Alistipes inops* and *Alistipes ssp*, whereas *Paracoccus denitrificans* and *Christensenellaceae R-7 group* spp. were found in *Orycte* sp. Although our primers were only designed to detect bacterial 16S rDNA, Archaeal rDNA represented 0.001% of reads in both species with *Methanobrevibacter arboriphilus* species being the only methanogen microorganisms detected.

**Fig 4:**
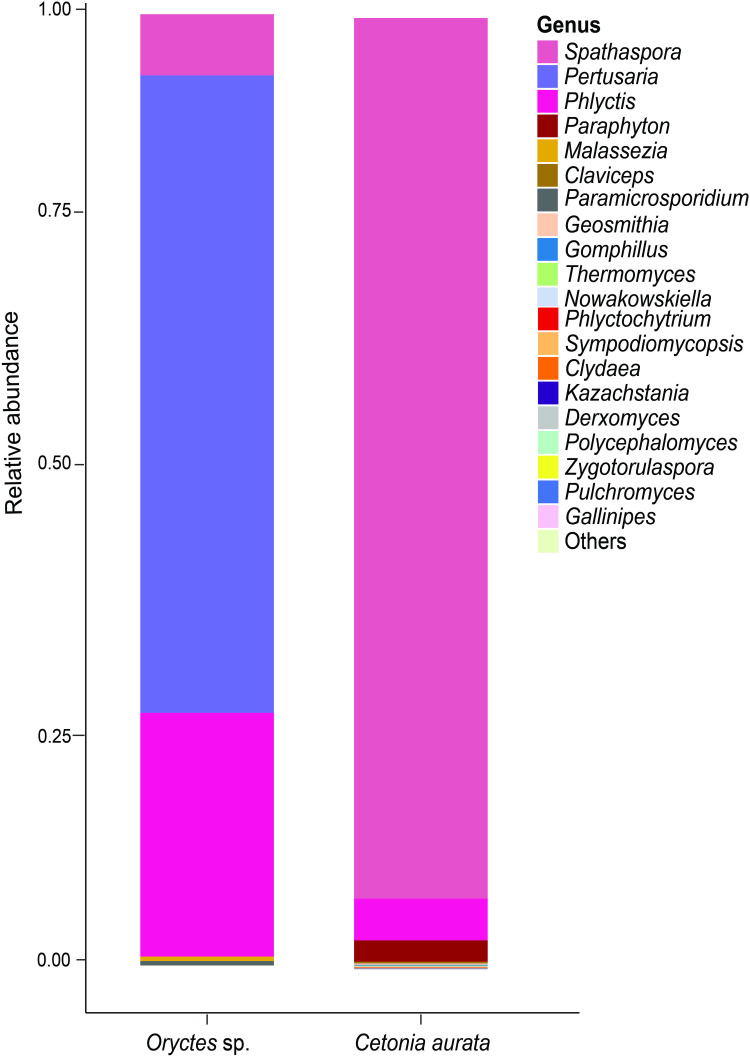
Taxonomic composition of fungal communities associated with Scarab beetle larvae. **Note.** Relative abundances of the most abundant ASVs are plotted at the genus level for *Oryctes* sp. and *C. aurata*. The “Others” group contained fungi that could not be classified at the genus level.

The fungal microbiota from the larvae were characterized from six samples by amplifying the 18S gene. A total of 94 taxa were identified, with the phylum Ascomycota accounting for nearly 99% of the total sequence reads. The phyla Basidiomycota, Chytridiomycota, and Rozellomycota accounted for the remaining sequence reads (1%) (S6 Table in S1 File). The class Lecanoromycetes (92.60%) exhibited greater dominance in *Oryctes* sp. larvae, whereas class Saccharomycetes (92.60%) was more prominent in *C. aurata* larvae (S7 Table in S1 File). The most dominant Ascomycetes in *Oryctes* sp. were from the genera *Pertusaria* and *Phlyctis*, while in *C. aurata*, it was from *Spathaspora* (Fig 4 and S8 Table in S1 File).

**Fig 5:**
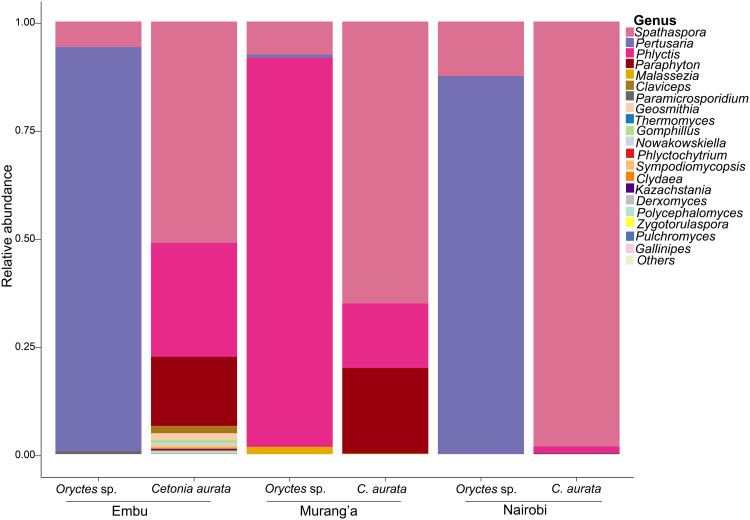
The comparative analysis of the taxonomic composition of fungal communities associated with *Oryctes* sp. and *C. aurata* from the same region conducted at the genus level. **Note.** The relative abundances of fungal ASVs were displayed using stacked bar plots for each beetle species. Fungi that could not be classified at the genus level were categorized under “Others”.

Similar to bacterial communities, differences in the relative abundance of fungal communities at the genus level were observed at allopatric sites. The genus *Paraphyton* dominated the gut microbiota of *C. aurata* beetle larvae from Embu and Murang’a, accounting for more than 16% of the total. *Phlyctis*, a lichenized fungus dominated the Murang’a *Oryctes* sp. larvae (Fig 5). At species level, *Spathaspora boniae* dominated in *C. aurata* with 92.6% prevalence, while *Pertusaria obruta* was dominant in *Oryctes* sp. with 67% (S9 Table in S1 File). Despite the differences in the relative abundances across the larval species, the specific shared gut microbes among the larvae were congruent.

### Unraveling the diversity of bacterial communities

**Fig 6:**
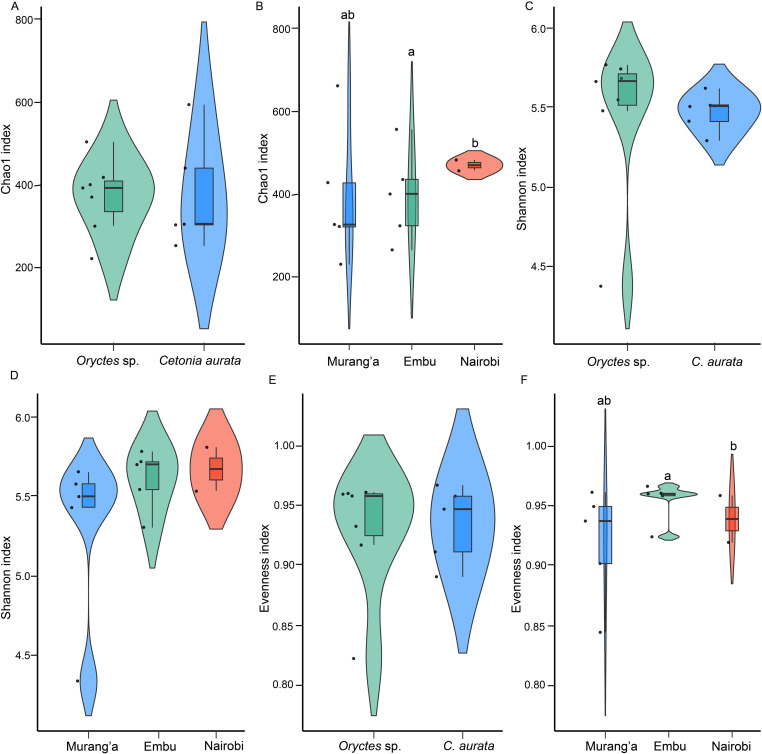
Alpha diversity in bacterial gut communities in scarab beetles collected from different sites. **Note.** Chao1 richness index estimates (6A, 6B), Shannon diversity index estimates (6C, 6D), and Evenness estimates (6E, 6F) for gut bacteria. Raincloud plots without letters on top are not significantly different. Raincloud plots combine an illustration of how the data is distributed (cloud) with rain which represents the jittered raw data. This is further combined with boxplots which have boxes representing the interquartile range (IQR) between the first and third quartiles. The horizontal line inside the box represents the median whereas the whiskers represent the lowest and highest values within the first and third quartiles, respectively.

Beetle larval phylogeny had no significant effects on species richness or evenness; P > 0.05, (Table 1). Analysis of *Oryctes* sp. larvae from Murang’a had a low number of observed OTUs (206), whereas those from other sites ranged from 301–545. Further, *C. aurata* (562.8) from Murang’a had a higher Chao1 value than the others, with *Oryctes* sp. (202.5) from the same region having the lowest. The samples collected from Nairobi and Embu exhibited similar species richness as compared to Murang’a ones (chi-squared = 7.87, df = 2, p-value = 0.04). The Shannon index estimations yielded a range of 4.3–5.7, with no significant variations across the groups (chi-squared = 6.90, df = 2, p-value = 0.07). Simpson evenness scores in both species ranged from 0.8 to 0.9. Additionally, samples from Nairobi and Embu exhibited comparable species evenness but varied significantly from the Murang’a: chi-squared = 8.10, df = 2, P value = 0.03 (Fig 6, Table 1). The gut fungal communities’ diversity of the two larvae analyzed cannot be compared since the quantity of fungal microbiota acquired from 18s might represent a relatively low proportion of the total gut microbial diversity (S1B Fig and S10 Table in S1 File).

### Interspecific variation and geographical site effect on scarab gut microbial communities

**Fig 7:**
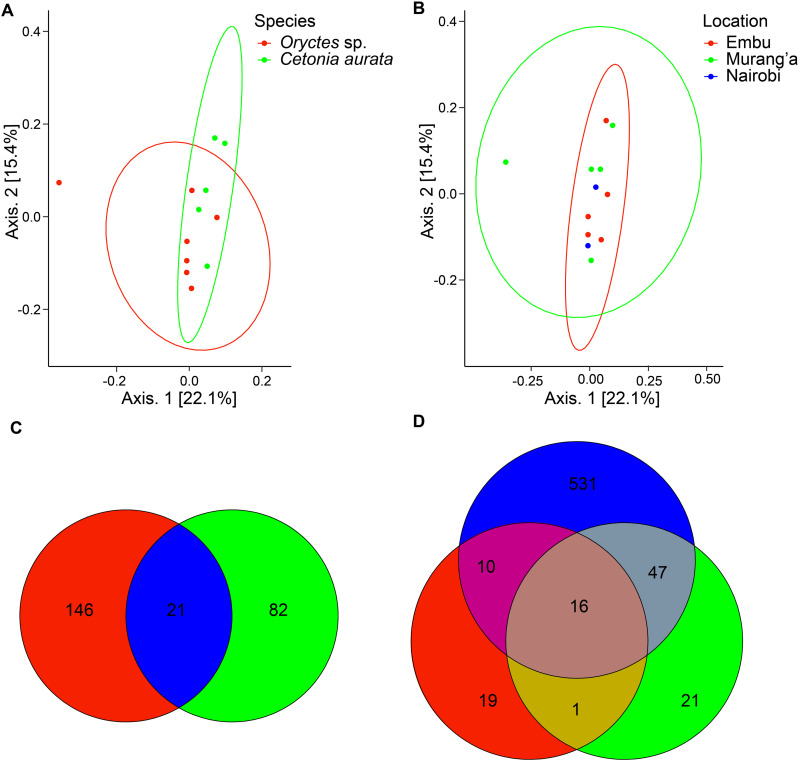
Beta diversity analysis of microbial communities associated with two beetle larvae species. **Note.** Unweighted UniFrac distance measurements in coprophagous beetle larvae between species (7A) and with regard to location (7B) derived from bacterial gut communities. Comparisons of the detritivores beetle larvae (7C) and location-wise (7D) using a Venn diagram to reveal the shared bacterial gut diversity. Values inside the Venn Diagrams indicate shared ASVs between the species and location wise.

Weighted UniFrac measures of diversity were used to compare the gut bacterial communities of detritivores beetle larvae, which account for the presence or absence of a specific bacterial sequence (ASVs), its phylogenetic relationship to other bacterial ASVs, and the abundance of the ASVs. To illustrate comparisons of the bacterial population between the two species and in relation to geographical collection location, a principal-coordinate analysis (PCoA) based on weighted UniFrac distances was utilized. The PCoA distances, using unweighted UniFrac distances, indicated that Axis 1 contributed 22.1% of the variation, whereas Axis 2 contributed 15.4% (Fig 7A and 7B). Here, *Oryctes* sp. had a subtle separation from *C. aurata* along axis 1 (Fig 7A) with Nairobi and Embu having a higher degree of intra-group clustering than Murang’a resulting from the differences in their relative abundances (Fig 7B). Furthermore, the geographic and host phylogeny effects, validated by pairwise PERMANOVA, considering Bray-Curtis distances, revealed that they did not influence the presence of the specific microbes (R^2^ = 0.11, P = 0.05) and (R^2^ = 0.16, P = 0.98), respectively (S10 Table in S1 File ).

Comparisons of core bacteria of the gut microbial community were done at the genus level to distinguish between bacteria that are stably associated with these scarabaeoid beetle larvae species. This was done by identifying bacterial communities with over 30% abundance at the genus level across the two larvae species. The larvae had a consistent core microbe of 21 species regardless of species (Fig 7C and S11 Table in S1 File). Regarding the location effect, only 16 bacterial genera were shared across all the sampling sites (Fig 7D and S12 Table in S1 File). The samples from Nairobi harbored 531 unique bacterial genera followed by Embu with 21 (Fig 7D). It was revealed that a large portion of the shared taxa were anerobic, sulfate-reducing bacteria from the family Desulfobacterota (*Desulfovibrionaceae*), Proteobacteria (Alphaproteobacteria), and fermentative bacteria Bacteroidetes (*Dysgonomonadaceae*).

### Functional analyses of the classified microbes

From the predicted PICRUSt2 results, the functional analysis of the gut microbiota revealed more abundant pathways (pathways with a total abundance below 200 across all samples were excluded) at primary levels like; generation of precursor metabolites, biosynthesis, assimilation/degradation/utilization and energy. Carbon and energy pathways that were highly expressed included protocatechuate degradation, lactose degradation, pyruvate fermentation to acetate and lactate II and the phosphate pentose along with processes such as fermentation, glycolysis, and the tricarboxylic acid (TCA) cycle. Regarding the nitrogen fixation process, metabolic pathways associated with amino acid uptake in bacteria such as aspartate and asparagine biosynthesis, glutamate and glutamine biosynthesis, lysine biosynthesis, threonine metabolism, and glycine betaine degradation pathways, were identified. Other pathways that aid in nitrogen fixation include myo-inositol degradation, formaldehyde assimilation, and purine and pyrimidine pathways. Fatty acid metabolism pathways such as lipid biosynthesis also play a significant role in supplying precursors known for symbiotic nitrogen fixation.

**Fig 8:**
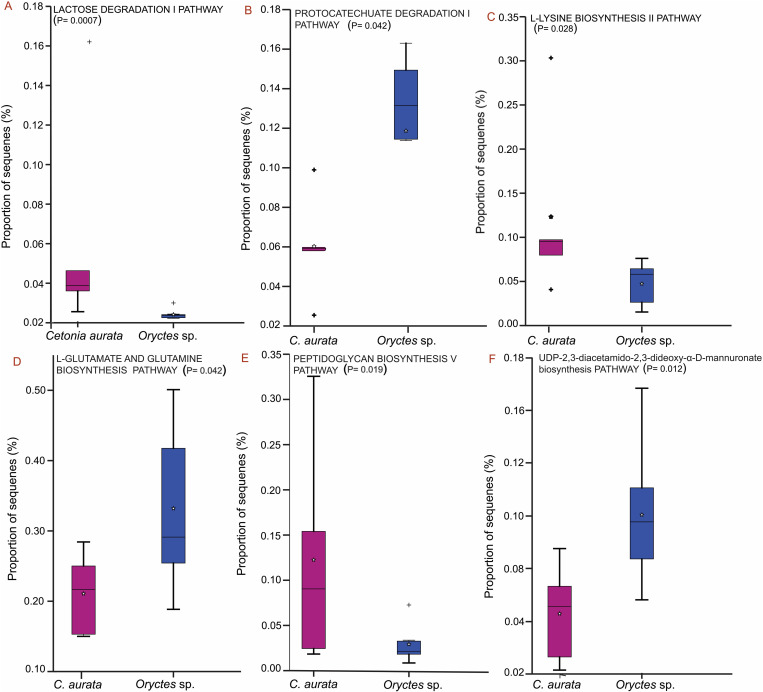
Box plots showing the significantly different most abundant KEGG pathways between *C. aurata* and *Oryctes* sp. larvae. **Note.** *C. aurata* had a higher proportion of sequences in lactose degradation I (8A), L-lysine biosynthesis II (8C) and peptidoglycan biosynthesis V (8E); while *Oryctes* sp. had a higher proportion of sequences in protocatechuate degradation I (8B), L-glutamate and L-glutamine biosynthesis (8D) and UDP-2,3-diacetamido-2,3-dideoxy-α-D-mannuronate biosynthesis(8F).

Super pathways such as demethylmenaquinol-6 biosynthesis II, Entner-Doudoroff pathway among others were also observed. All these categories were distributed among the saprophagous beetle larvae (S3 Fig in S1 File). A significant difference (P < 0.05) was found in the following KEGG pathways; *C. aurata* microbiota had a higher abundance in lactose degradation I (LACTOSECAT-PWY), L-lysine biosynthesis II (PWY-2941), peptidoglycan biosynthesis V (PWY-6470) while *Oryctes* sp. was more enriched in protocatechuate degradation I (P184-PWY), L-glutamate and L-glutamine biosynthesis (PWY-5505), UDP-2,3-diacetamido-2,3-dideoxy-α-D-mannuronate biosynthesis (PWY-7090) (Fig 8).

## Discussion

Gut microbiota represents a complex and diversified system in which thousands of microbial species dwell. These microbes, particularly in dung beetles, evolve with the host and serve a vital role in the digestive systems of the insects since they rely on hard to digest food sources or limited nutrients [36,37]. In this study, the microbial communities of two closely related saprophagous beetle larvae were characterized to determine whether geographical location and host phylogeny had influence on the microbial composition. The main bacterial phyla; firmicutes, protobacteria and Bacteroidetes found in the intestinal tract of many animals and insects are also present in the two beetle larvae. This is in agreement with the results reported from the larvae of red palm weevil, *Rhynchophorus ferrugineus* Oliver, and the date palm root borer, *Oryctes Agamemnon* as the most prevalent phyla groups in their gut microbiota [38,39].

Although previous studies suggest that there is correlation between bacterial diversity with diet, complex environs and the beetle phylogeny [8,40,41], differences were observed in the relative abundance of the microbes. The main variation in the gut microbes between the two larvae was in relative abundance of the ASVs (and genera) rather than the presence or absence of particular microbes. Similar results have been reported in other insects like the burying dung beetles – *Euoniticellus intermedius* and *E. triangulatus* [42], the butterfly *Heliconius erato* [43], dung Beetle *Copris incertus* [44].

The variation in the allopatric larvae might be attributed to differences in abiotic conditions within the host geographical location, which could directly influence host phenotype. For instance, aspects of host diet such as fiber content [45], salinity [46] and prey diversity [47] might vary spatially, altering the gut community composition by favoring some microbes over others. Further, temperature and humidity changes are competitive dynamics among the gut microbiota by modifying the relative abundance of microbial community members [48]. Moreover, gut-specialized microbes frequently have lower thermal tolerances than their hosts or may confer distinct selection benefits to the host depending on the temperature. Thus, the observed differences in the relative abundances of gut microbiota in the allopatric two scarab beetle larvae could be attributed to variations in vegetation cover across the three counties, which influence the composition of the dung. Apart from the differences in relative abundances, the specific gut microbes were shared between the two larvae. The striking resemblance between the larvae might be attributed to the fact that the microbes were acquired from their surroundings at some point during their existence, regardless of whether the transmission from one generation of hosts to the next was vertical or horizontal [49]. Moreover, the host phylogeny did not influence the gut microbiota composition. Few or no fungal species identified in this current study have also been reported in the gut microbiomes of other beetles such as the Pachysoma MacLeay desert dung beetles [8].

### Funcional role of in waste management and bioremediation

The variety and complexity of insect-bacteria interactions provide significant possibilities for biotechnological applications. Functional analyses of the dung beetle larvae in this study showed that the identified microbial communities had various metabolic functions valuable to the host. However, most of the microbes are uncultured hence the predicted metabolic functions are only rough estimations based on available reference data. For instance, the prevalent presence of cellulase-producing strains within families like Bacillaceae, Bacteroidetes, Clostridiaceae, Christensenellaceae and Pseudomonadaceae underscores their crucial contribution to breaking down lignocellulosic biomass [50]. This is particularly relevant in the context of rapid urbanization and population growth, alongside inadequate domestic waste management, which leads to uncontrolled waste generation and accumulation, increased greenhouse gas emissions and accumulation, especially in low-income countries [51]. The prevailing waste management strategies are neither environmentally eco-friendly nor economically sustainable [52]. However, beetle gut microbes perform complex metabolic processes on lignocellulosic material offer a glimmer of hope. *Bacillus* species, *Pseudomonas mosselii* and the putative symbiont *Proteiniphilum* have known lignocellulosic biodegradation activities as previously reported in other insects [16,53–55]. Notably, the TCA and protocatechuate degradation pathways were more expressed in the beetle larvae from Nairobi County that actively feed on green composting trash, highlighting their utility as bioreactors for lignocellulose degradation in domestic waste.

Regarding archaea, methanogenic archaea from the phylum Euryarchaeota have been found in other beetles, millipedes, termites, and cockroaches [56,57]. Few or no fungal species identified in this current study have also been reported in the gut microbiomes of other beetles such as the Pachysoma MacLeay desert dung beetles [58]. The fungi (Chytridiomycota) and yeast (*Spathaspora*) microbes from the insect gut microbiota break the lignin-cellulose linkages [59,60]. Yeasts and some actinomycetes also aid in the degradation of hydrolysable tannins, phenolic polymer produced by higher plants [61]. Other present pathways involved in carbon assimilation include Leloir pathway: which involves galactose sugar degradation to biomass and energy in the presence of yeast strains such as *Candida albicans* and baker’s yeast *Saccharomyces cerevisiae* [62]. Contrary to our results, the two strains were not present implying that other Saccharomycetes yeasts like *Spathaspora boniae* present may perform similar roles. These microbes play a significant role in promoting at eco-friendly waste management and biodegradation of materials valuable for recycling.

In nitrogen-limited environments, biological nitrogen fixation is performed by diazotrophs, while auxotrophic hydrocarbon-degrading bacteria rely on them to provide essential nutrients. This synergistic mechanism helps increase the degradation rate of pollutants [63]. The presence of nitrogen-fixing pathways in both hydrocarbon-degrading bacteria (Gammaproteobacteria) and diazotrophs (Bacillus and Clostridium) is notable. This supports the use of larval microbiota in pollutant degradation in nitrogen-limited environments, such as farmland soil contaminated with persistent organic pollutants (POPs). Organochlorine pesticides such as dichlorodiphenyltrichloroethane (DDT) and benzene hexachloride (BHC) use in agriculture have contributed significantly to increase in food production. Even though their use was banned over 45 years ago, they pose a threat to human health and food security due to their lipophilic nature, low degradation rates and ability bioaccumulate in food chain [64]. To reduce environmental pollution due to POPs, a biological and non -chemical approach like understanding the biochemical metabolic pathways in microbes in order to design bioremediation strategies can be exploited.

Under anaerobic conditions, denitrifying, sulfate and carbonate bacteria prevail against methanogens. Sulfate reducing bacteria are versatile and can use wide electron acceptors such as metals, sulfur derivatives and halogenated organic compounds [65]. One the Desulfobacterota bacteria has been documented to be capable of de-halogenating chlorinated aromatic compounds using a hybrid bioinorganic catalyst [66]. Further, Bacilli, Clostridia, Gammaprotobacteria, Actinobacteria and Alphaprotobacteria aided in the degradation of dioxin, pentachloronitrobenzene and lindane [67,68]. Microbial remediation is a green, sustainable and nature-based waste management process that leverages microbes’ ability to degrade organic pollutants. Introducing specific microbes to polluted soils or environments could help reclaim them.

The role of insects’ microbes in ecological cycles such as the carbon cycle cannot be over emphasized. Carbon (IV) oxide might be assimilated by reductive Krebs cycle which was observed across all larvae species and mainly associated with *Desulfovibrio* bacteria [69]. Overall, the presence of these chemolithoautotrophy pathways denotes that these microbes might play a key role in carbon cycle. This implies that bacteria capable of capturing and storing carbon (IV) oxide may be viable candidates for use as biologically lowering CO_2_ fingerprints in the atmosphere, as well as manufacturing platforms for a variety of CO_2_-derived bio-products.

### Functional role of gut microbiota for food and feed and as targets for bio-therapeutic interventions

Currently, there is a growing consumer interest in healthier food options aimed at maintaining wellbeing of the population. By 2029, this shift towards wellness is anticipated to elevate the probiotics segment within functional foods, projecting an estimated market value increase to USD 105.7 billion [70]. The incorporation of microorganisms into foods not only presents significant technological benefits but also offers therapeutic advantages, underscoring their holistic role in advancing food science and health. On the other hand, *Bacillus* species exhibit remarkable resilience by thriving under adverse conditions, including the gastrointestinal tracts of various organisms other than insects, due to their capacity to widely sporulate [71]. This resilience positions them as fascinating targets probiotic research in functional foods field.

Synergistic effects observed from combining diverse *Bacillus* strains enhance the production of functional metabolites contributing to the maintenance of intestinal homeostasis in hosts [72]. These functional metabolites, when accumulated through *in vitro* fermentation and subsequently utilized *in vivo*, present enhanced benefits [73,74]. Consequently, *C. aurata* larvae emerged as a promising reservoir of diverse *Bacillus* strains that could be advanced for probiotics research in nutrition. Furthermore, these larvae hold potential as prebiotics to supplement the nutritional profile of livestock such as pigs, chickens, and fish. The escalating unsustainability in the rearing of these animals, exacerbated by the pervasive use of antibiotics and the looming threat of antimicrobial resistance, underscores the significance of exploring alternative nutritional strategies [75]. Moreover, in the management of intestinal diseases, probiotics aid in regulating the intestinal microbiota and alleviating inflammation. Recent research involving the synergistic effect of probiotics containing *Lactococcus* and *Enterococcus* bacterial strains found that they had an inhibitory effect on intestinal colitis in mice induced by *Citrobacter* [76]. Leveraging *C. aurata* larvae as a prebiotic source could offer a sustainable solution, mitigating reliance on antimicrobials and enhancing the nutritional management of livestock.

A new generation probiotic organism, the Christensenellaceae group, also identified in this study was initially identified in healthy human feces and recently isolated from Coconut rhinoceros beetle (*O. rhinoceros*) and Japanese beetles (*Popillia japonica*) [12,50]. The Christensenella family encompasses commensal bacteria with transmissible properties that exhibit mutual interactions with other heritable microbes. Their relative abundance has been found to be positively correlated with the lean host phenotype, associated with a low body mass index [77]. This group has been shown to help in the management of metabolic diseases, including inflammatory bowel disease, obesity, and type 2 diabetes, by counteracting microbiota dysbiosis and altering host metabolism. Microbial dysbiosis, characterized by a decrease in beneficial bacteria, is associated with metabolic disorders and contributes to higher mortality rates from these diseases [77]. To preserve the balance of the host’s gut microbiota, isolating the Christensenellaceae group from scarabaeoid larvae mighty present a promising option for novel biotherapeutic approaches either through oral administration as a probiotic or fecal microbiota transplantation.

Other microbes reported with probiotic activity are *Alistipes inops* and *Alistipes ssp* previously isolated from human microbiome/fecal matter not insects [78]. Despite being reported in our present investigation, they may be facultative symbionts in insects since the dung from each county included mixed fiber dietary items such as legumes and whole grains, which are known sources of these bacteria.

Scarab beetle larvae spend their whole lives in dung which contains pathogenic microbes released from indigestible material excreted by mammals’ gastrointestinal tract [79]. Some colonies of Firmicutes, Proteobacteria and Actinobacteria obtained from the oral secretions of the bark beetle *Dendroctonus rufipennis* were shown to significantly inhibit the development of antagonistic fungi [80]. These symbiotic relationships could be key in safeguarding either the insect host or its nutritional supplies from predators and parasitoids. The larval samples from Nairobi, unlike those in the other regions, live in marshy compost piles prone to human pollution and harbored many unique bacterial species. These larvae could have evolved ontogenetically, and host the many unique microbes to help them in defense, digestion or metabolic processes while in their adverse environs.

**Fig 9:**
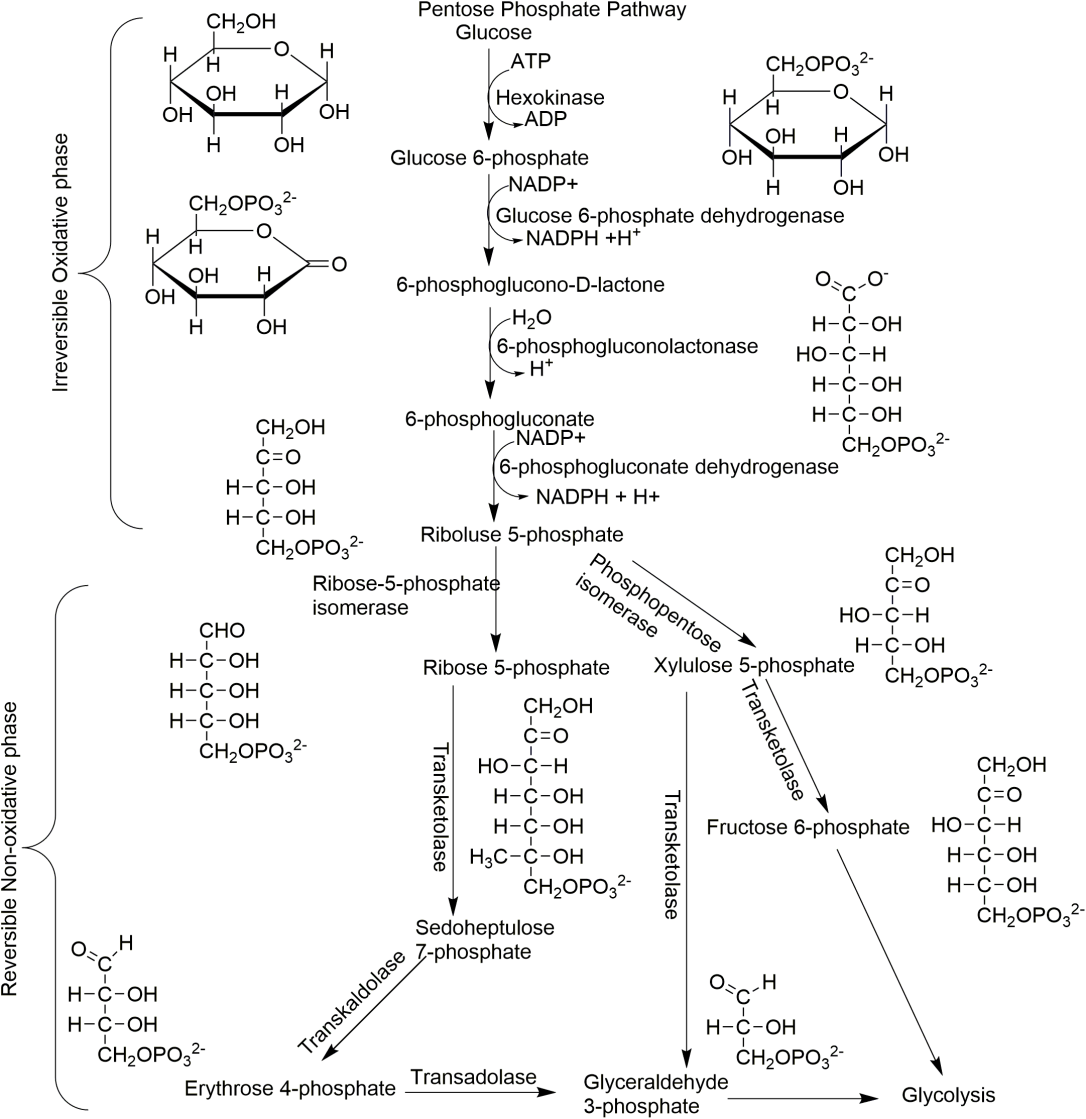
Utilization of glucose using the pentose phosphate pathway in scarabaeoid gut community. **Note**. The reactions are catalyzed by bacteria or yeasts present in the gut of the beetles. Glucose released from cellulose can be converted into xylulose 5-phosphate and eventually to pyruvate. Nicotinamide adenine dinucleotide phosphate (NADPH), nicotinamide adenine dinucleotide (NAD), ATP (Adenosine Triphosphate), ADP and (Adenosine Diphosphate).

In terms of metabolic pathways, the pentose phosphate pathway (PPP), glycolysis, and the tricarboxylic acid cycle were present, which help in supplying biosynthetic intermediates for anabolic and catabolic processes. The PPP (Fig 9) is a major pathway for glucose metabolism, which involves an irreversible oxidative phase and reversible non-oxidative phases. The oxidative phase involves the conversion of glucose-6-phosphate, a product of glycolysis, to 6-phosphogluconate using the glucose-6-phosphate dehydrogenase enzyme, resulting in the creation of nicotinamide adenine dinucleotide phosphate (NADPH) molecule. As 6-phosphogluconate is converted into ribulose-5-phosphate, another molecule of NADPH is created.

On the other hand, the non-oxidative phase begins with the conversion of ribulose-5-phosphate into xylulose 5-phosphate or ribose 5-phosphate. The other reactions involve transketolase or transaldolase enzymes, leading to the formation of sedoheptulose 7-phosphate, erythrose 4-phosphate (used in the formation of amino acids), and fructose-6-phosphate (which returns back to glycolysis) [81]. Sedoheptulose 7-phosphate is used by gram-negative bacteria to create a lipopolysaccharide layer, which is a metabolic adaptation designed to evade host stressors, leading to antibiotic resistance. Disrupting the PPP process not only affects the energy supply to these multi-drug resistant bacteria but also directly impacts biofilm formation and other resistance mechanisms [82]. This in turn affects their pathogenicity and could aid in combating drug resistance.

## Conclusion

A comprehensive analysis of intestinal microbes associated with scarab beetle larvae was investigated, and their functional implications were predicted. The gut microbiota of the two scarabaeoid larvae was found to be similar to that reported in other insects, including their plausible biological functions. However, Alphaproteobacteria (29.60%) and Actinobacteria (8.60%) emerged as the predominant bacterial classes in *Oryctes* sp., contrasting sharply with *C. aurata*, which exhibited a notable dominance of Bacteroidia (32.50%), Bacilli (22.40%), and Clostridia (19.20%). This diversity underscores the distinct microbial communities associated with each species. Further investigation is needed to determine whether variations in community composition are influenced by bacterial communities present in the feed. Nevertheless, host phylogeny did not influence the microbiota composition for both species. Metagenomics and functional predictions uncovered the mechanisms underlying cellulose and nitrogen degradation in scarabaeoids. Our results suggest that the degradation of cellulose and nitrogen waste could be successfully driven by the bacterial families Bacillaceae, Bacteroidetes, Clostridiaceae, Christensenellaceae, and Pseudomonadaceae. Additionally, the core bacterium Desulfobacterota known for its sulfate-degrading capabilities and *Nocardioides* sp. could aid in the degradation of persistent organic pollutants, which are a major global concern. Clearly, various factors influence the composition of the gut microbiota, and future investigations are needed to contribute to a better understanding of variations in beetles. The functional predictions not only give an improved understanding of the intricate relationship between gut microbes and the host, but also provide a theoretical basis for future biotechnological applications in environmental conservation, food and drug discovery industries.

## Supporting information

S1 File The raw reads of the sequences used in this study for all samples are available upon request. Supporting information has been provided (Tables S1- S11 and Figures S1-S3).
(ZIP)
