# Supplementary material for: Elucidating the functions of gut microbiota from two edible dung beetle species: Implications for waste management and food industry
Source: PLoS One. 2025 Jun 25;20(6):e0325756. doi: 10.1371/journal.pone.0325756 (PMC12193771; doi:10.1371/journal.pone.0325756)
Supplement: S1 File — Supporting information has been provided (Tables S1- S11 and Figures S1-S3). (ZIP) [file pone.0325756.s001.zip › Suppl/Supporting Information.docx]

**Elucidating the functions of gut microbiota from two edible dung beetle species: implications for waste management and food industry**

Mwanza Sylviah Syombua ^1,2^, Cynthia M. Mudalungu^1,3*^, JohnMark Makwatta^1^, James Kabii^1^, Njogu M. Kimani^2^, Chrysantus M. Tanga^1*^

^1^Department of behavioral and chemical ecology, International Centre of Insect Physiology and Ecology (*icipe*), Kasarani, Nairobi, Kenya.

^2^Department of Physical sciences, University of Embu, Embu, Kenya.

^3^Department of Chemistry and Material science, Technical University of Kenya, Haile Selassie Avenue, Nairobi, Kenya.

**S1 Table:** Total number of rarified fungal sequences and diversity of fungal communities in both Scarabaeoid beetle larvae (n = 5), sequenced as a pool

| **Species** | **Sample ID** | **Collection locality** | **Total no. of rarefied fungal sequences** |  | **observed** | **Chao1** | **Simpson** | **Shannon** | **Evenness** |
| --- | --- | --- | --- | --- | --- | --- | --- | --- | --- |
| Oryctes sp. | DBA1 | Murang’a | 151 |  | 3 | 3 | 0.23 | 0.46 | 0.42 |
| Oryctes sp. | DBB1 | Embu | 5444 |  | 5 | 6 | 0.69 | 1.32 | 0.55 |
| Oryctes sp. | DBR1 | Nairobi | 11211 |  | 2 | 2 | 0.15 | 0.29 | 0.54 |
| *C. aurata* | FBB4 | Embu | 25692 |  | 11 | 16.25 | 0.76 | 1.91 | 0.52 |
| *C. aurata* | FBA4 | Murang’a | 23518 |  | 4 | 4 | 0.46 | 0.88 | 1 |
| *C. aurata* | FBR2 | Nairobi | 100436 |  | 3 | 4 | 0.16 | 0.34 | 0.39 |

**S2 Table**: Percentage abundance of the bacterial ASVs at the Phylum level for both species

| **Phylum** | **Oryctes sp.** | ***C. aurata*** |
| --- | --- | --- |
| Firmicutes | 25.30% | 42.10% |
| Bacteroidota | 15.30% | 32.50% |
| Proteobacteria | 35.00% | 10.20% |
| Actinobacteriota | 11.40% | 9.60% |
| Desulfobacterota | 7.40% | 2.60% |
| Patescibacteria | 0.80% | 0.90% |
| Planctomycetota | 2.20% | 0.70% |
| Deferribacterota | 0.50% | 0.40% |
| Myxococcota | 0.10% | 0.30% |
| Acidobacteriota | 0.30% | 0.20% |
| Deinococcota | 0.40% | 0.20% |
| Verrucomicrobiota | 0.80% | 0.20% |

**S3 Table:** Percentage abundance of the bacterial ASVs at the Class level for both species

| **Class** | **Oryctes sp.** | ***C. aurata*** |
| --- | --- | --- |
| Alphaproteobacteria | 29.60% | 4.50% |
| Clostridia | 17.50% | 19.20% |
| Bacteroidia | 15.30% | 32.50% |
| Actinobacteria | 8.60% | 8.40% |
| Bacilli | 7.40% | 22.40% |
| Desulfovibrionia | 7.40% | 2.60% |
| Gammaproteobacteria | 5.40% | 5.70% |
| Planctomycetes | 2.20% | 0.70% |
| Acidimicrobiia | 2.00% | 0.60% |
| Saccharimonadia | 0.80% | 0.90% |
| Verrucomicrobiae | 0.80% | 0.20% |
| Coriobacteriia | 0.50% | 0.40% |
| Deferribacteres | 0.50% | 0.40% |
| Deinococci | 0.40% | 0.20% |
| Thermoleophilia | 0.40% | 0.30% |
| Desulfitobacteriia | 0.20% | 0.00% |
| Acidobacteriae | 0.10% | 0.10% |
| Blastocatellia | 0.10% | 0.00% |
| Desulfotomaculia | 0.10% | 0.30% |
| Endomicrobia | 0.10% | 0.00% |
| Syntrophomonadia | 0.10% | 0.10% |
| Vicinamibacteria | 0.10% | 0.10% |

**S4 Table**: Relatively abundant bacterial classes in the Scarab beetle larvae (30 most abundant genera)

| **Genus** | **Oryctes sp.** | ***C. aurata*** |
| --- | --- | --- |
| *Paracoccus* | 17.60% | 0.30% |
| *Christensenellaceae R-7 group* | 10.20% | 13.70% |
| *Proteiniphilum* | 9.30% | 5.10% |
| *Desulfovibrio* | 7.40% | 2.60% |
| *Ensifer* | 5.10% | 2.20% |
| *Alistipes* | 2.30% | 23.90% |
| *Ochrobactrum* | 2.00% | 0.10% |
| *Kocuria* | 1.80% | 0.20% |
| *Jeotgalibaca* | 1.70% | 0.00% |
| *Paludibacter* | 1.50% | 0.00% |
| *Tyzzerella* | 1.30% | 0.80% |
| *Bacillus* | 1.20% | 9.50% |
| *Turicibacter* | 1.10% | 0.50% |
| *Iamia* | 1.10% | 0.30% |
| *Cellulomonas* | 1.10% | 0.60% |
| *Aminobacter* | 1.10% | 0.00% |
| *Pseudoxanthomonas* | 1.00% | 0.20% |
| *Papillibacter* | 0.80% | 0.10% |
| *Alkalibacterium* | 0.80% | 0.00% |
| *Ilumatobacter* | 0.70% | 0.30% |
| *Nocardioides* | 0.60% | 0.50% |
| *Rhodococcus* | 0.50% | 0.40% |
| *Mucispirillum* | 0.50% | 0.40% |
| *Candidatus Soleaferrea* | 0.40% | 1.10% |
| *Pseudomonas* | 0.20% | 2.20% |
| *Bacteroides* | 0.20% | 0.90% |
| *Lysinibacillus* | 0.10% | 1.40% |
| *Enterococcus* | 0.10% | 7.20% |
| *Dysgonomonas* | 0.10% | 2.20% |
| *Lactococcus* | 0.00% | 1.30% |

**S5 Table**: Percentage abundance of bacterial species identified from both species

| **Species** | **Oryctes sp.** | ***C. aurata*** |
| --- | --- | --- |
| *Paracoccus denitrificans* | 17.80% | 0.10% |
| *Christensenellaceae R-7 group spp* | 10.00% | 13.80% |
| *Proteiniphilum spp* | 7.20% | 4.10% |
| *Desulfovibrio spp* | 7.10% | 2.70% |
| *Ensifer spp* | 4.20% | 1.70% |
| *Alistipes spp* | 2.10% | 14.30% |
| *Kocuria rosea* | 1.90% | 0.00% |

**S6 Table**: Percentage abundance of the fungal ASVs at the Phylum level for both species

| **Phylum** | **Oryctes sp.** | ***C. aurata*** |
| --- | --- | --- |
| Ascomycota | 99.10% | 99.80% |
| Basidiomycota | 0.50% | 0.10% |
| Chytridiomycota | 0.00% | 0.20% |
| Rozellomycota | 0.50% | 0.00% |

**S7 Table**: Percentage abundance of the fungal ASVs at the Class level for both species

| **Class** | **Oryctes sp.** | ***C. aurata*** |
| --- | --- | --- |
| Sordariomycetes | 0.00% | 0.30% |
| Saccharomycetes | 6.40% | 92.60% |
| Rozellomycota_cls_Incertae_sedis | 0.50% | 0.00% |
| Malasseziomycetes | 0.50% | 0.00% |
| Lecanoromycetes | 92.40% | 4.50% |
| Eurotiomycetes | 0.20% | 2.30% |
| Cladochytriomycetes | 0.00% | 0.10% |
| Chytridiomycetes | 0.00% | 0.10% |

**S8 Table**: Percentage relative abundance of fungal genera in both Scarab beetle larvae species

| **Genus** | **Oryctes sp.** | ***C. aurata*** |
| --- | --- | --- |
| *Pertusaria* | 67.00% | 0.00% |
| *Phlyctis* | 25.60% | 4.40% |
| *Spathaspora* | 6.40% | 92.60% |
| *Malassezia* | 0.50% | 0.00% |
| *Paramicrosporidium* | 0.50% | 0.00% |
| *Claviceps* | 0.00% | 0.20% |
| *Geosmithia* | 0.00% | 0.20% |
| *Gomphillus* | 0.00% | 0.10% |
| *Nowakowskiella* | 0.00% | 0.10% |
| *Paraphyton* | 0.00% | 2.30% |
| *Phlyctochytrium* | 0.00% | 0.10% |

**S9 Table**: Percentage abundance of fungi species identified from both species

| **Species** | **Oryctes sp.** | ***C. aurata*** |
| --- | --- | --- |
| *Claviceps panicoidearum* | 0.00% | 0.20% |
| *Clydaea vesicula* | 0.00% | 0.00% |
| *Derxomyces spp* | 0.00% | 0.00% |
| *Gallinipes pseudodichotomus* | 0.00% | 0.00% |
| *Geosmithia eupagioceri* | 0.00% | 0.20% |
| *Gomphillus calycioides* | 0.00% | 0.10% |
| *Kazachstania aquatica* | 0.00% | 0.00% |
| *Malassezia vespertilionis* | 0.50% | 0.00% |
| *Nowakowskiella spp* | 0.00% | 0.10% |
| *Paramicrosporidium saccamoebae* | 0.50% | 0.00% |
| *Paraphyton spp* | 0.00% | 2.30% |
| *Pertusaria obruta* | 67.00% | 0.00% |
| *Phlyctis speirea* | 25.60% | 4.40% |
| *Phlyctochytrium arcticum* | 0.00% | 0.10% |
| *Polycephalomyces tomentosus* | 0.00% | 0.00% |
| *Pulchromyces fimicola* | 0.00% | 0.00% |
| *Spathaspora boniae* | 6.40% | 92.60% |
| *Sympodiomycopsis spp* | 0.00% | 0.00% |
| *Thermomyces lanuginosus* | 0.00% | 0.10% |
| *Zygotorulaspora dagestanica* | 0.00% | 0.00% |

**S10 Table:** PERMANOVA results for weighted UNIFRAC matrices of intestinal bacterial genera

|  | **Df** | **MS** | **F** | **R2** | **Pr** |
| --- | --- | --- | --- | --- | --- |
| Location | 2 | 0.541162 | 1.1941 | 0.1066689 | 0.0592 |
| Species | 1 | 0.401639 | 0.8451 | 0.158108 | 0.9799 |

**S11 Table**: Common shared ASVs between species

| ASV_135 | Bacteria | Desulfobacterota | Desulfovibrionia | Desulfovibrionales | Desulfovibrionaceae | *Desulfovibrio* |
| --- | --- | --- | --- | --- | --- | --- |
| ASV_176 | Bacteria | Desulfobacterota | Desulfovibrionia | Desulfovibrionales | Desulfovibrionaceae | *Desulfovibrio* |
| ASV_205 | Bacteria | Desulfobacterota | Desulfovibrionia | Desulfovibrionales | Desulfovibrionaceae | *Desulfovibrio* |
| ASV_170 | Bacteria | Desulfobacterota | Desulfovibrionia | Desulfovibrionales | Desulfovibrionaceae | *Desulfovibrio* |
| ASV_24 | Bacteria | Desulfobacterota | Desulfovibrionia | Desulfovibrionales | Desulfovibrionaceae | *Desulfovibrio* |
| ASV_546 | Bacteria | Desulfobacterota | Desulfovibrionia | Desulfovibrionales | Desulfovibrionaceae | *Desulfovibrio* |
| ASV_108 | Bacteria | Desulfobacterota | Desulfovibrionia | Desulfovibrionales | Desulfovibrionaceae | *Desulfovibrio* |
| ASV_86 | Bacteria | Desulfobacterota | Desulfovibrionia | Desulfovibrionales | Desulfovibrionaceae | *Desulfovibrio* |
| ASV_110 | Bacteria | Desulfobacterota | Desulfovibrionia | Desulfovibrionales | Desulfovibrionaceae | *Desulfovibrio* |
| ASV_71 | Bacteria | Desulfobacterota | Desulfovibrionia | Desulfovibrionales | Desulfovibrionaceae | *Desulfovibrio* |
| ASV_76 | Bacteria | Desulfobacterota | Desulfovibrionia | Desulfovibrionales | Desulfovibrionaceae | *Desulfovibrio* |
| ASV_130 | Bacteria | Desulfobacterota | Desulfovibrionia | Desulfovibrionales | Desulfovibrionaceae | *Desulfovibrio* |
| ASV_127 | Bacteria | Desulfobacterota | Desulfovibrionia | Desulfovibrionales | Desulfovibrionaceae | *Desulfovibrio* |
| ASV_147 | Bacteria | Desulfobacterota | Desulfovibrionia | Desulfovibrionales | Desulfovibrionaceae | *Desulfovibrio* |
| ASV_151 | Bacteria | Desulfobacterota | Desulfovibrionia | Desulfovibrionales | Desulfovibrionaceae | *Desulfovibrio* |
| ASV_203 | Bacteria | Desulfobacterota | Desulfovibrionia | Desulfovibrionales | Desulfovibrionaceae | *Desulfovibrio* |
| ASV_102 | Bacteria | Desulfobacterota | Desulfovibrionia | Desulfovibrionales | Desulfovibrionaceae | *Desulfovibrio* |
| ASV_134 | Bacteria | Desulfobacterota | Desulfovibrionia | Desulfovibrionales | Desulfovibrionaceae | *Desulfovibrio* |
| ASV_442 | Bacteria | Desulfobacterota | Desulfovibrionia | Desulfovibrionales | Desulfovibrionaceae | *Desulfovibrio* |
| ASV_129 | Bacteria | Desulfobacterota | Desulfovibrionia | Desulfovibrionales | Desulfovibrionaceae | *Desulfovibrio* |
| ASV_53 | Bacteria | Desulfobacterota | Desulfovibrionia | Desulfovibrionales | Desulfovibrionaceae | *Desulfovibrio* |

**S12 Table**: Common shared ASVs across the sampling sites

| ASV_136 | Bacteria | Proteobacteria | Alphaproteobacteria | Rhizobiales | Rhizobiaceae | *Ensifer* |
| --- | --- | --- | --- | --- | --- | --- |
| ASV_174 | Bacteria | Proteobacteria | Alphaproteobacteria | Rhizobiales | Rhizobiaceae | *Ensifer* |
| ASV_197 | Bacteria | Proteobacteria | Alphaproteobacteria | Rhizobiales | Rhizobiaceae | *Ensifer* |
| ASV_48 | Bacteria | Proteobacteria | Alphaproteobacteria | Rhizobiales | Rhizobiaceae | *Ensifer* |
| ASV_159 | Bacteria | Proteobacteria | Alphaproteobacteria | Rhizobiales | Rhizobiaceae | *Ensifer* |
| ASV_258 | Bacteria | Bacteroidota | Bacteroidia | Bacteroidales | Dysgonomonadaceae | *Proteiniphilum* |
| ASV_173 | Bacteria | Proteobacteria | Alphaproteobacteria | Rhizobiales | Rhizobiaceae | *Ensifer* |
| ASV_155 | Bacteria | Proteobacteria | Alphaproteobacteria | Rhizobiales | Rhizobiaceae | *Ensifer* |
| ASV_65 | Bacteria | Proteobacteria | Alphaproteobacteria | Rhizobiales | Rhizobiaceae | *Ensifer* |
| ASV_181 | Bacteria | Proteobacteria | Alphaproteobacteria | Rhizobiales | Rhizobiaceae | *Ensifer* |
| ASV_177 | Bacteria | Proteobacteria | Alphaproteobacteria | Rhizobiales | Rhizobiaceae | *Ensifer* |
| ASV_132 | Bacteria | Proteobacteria | Alphaproteobacteria | Rhizobiales | Rhizobiaceae | *Ensifer* |
| ASV_175 | Bacteria | Proteobacteria | Alphaproteobacteria | Rhizobiales | Rhizobiaceae | *Ensifer* |
| ASV_125 | Bacteria | Proteobacteria | Alphaproteobacteria | Rhizobiales | Rhizobiaceae | *Ensifer* |
| ASV_208 | Bacteria | Proteobacteria | Alphaproteobacteria | Rhizobiales | Rhizobiaceae | *Ensifer* |
| ASV_79 | Bacteria | Proteobacteria | Alphaproteobacteria | Rhizobiales | Rhizobiaceae | *Ensifer* |

**S13 Table**: Overview of the beetle larvae specimens examined in this study. Table includes species identity, collection locality and the number of bacterial sequence reads for individual samples after filtering, denoising, merging, tabling as ASVs and removal of chimeras.

| **Species** | **Sample ID** | **Collection locality** | **Input** | **Filtered** | **Denoised** | **Merged** | **Tabled** | **Nonchim** |
| --- | --- | --- | --- | --- | --- | --- | --- | --- |
| Oryctes sp. | DBA1 | Murang’a | 85079 | 73722 | 72617 | 69951 | 69951 | 32310 |
| Oryctes sp. | DBB1 | Embu | 73291 | 62056 | 60994 | 55625 | 55625 | 35695 |
| Oryctes sp. | DBA2 | Murang’a | 70605 | 67068 | 63763 | 56253 | 56253 | 32285 |
| Oryctes sp. | DBB2 | Embu | 53892 | 51342 | 48140 | 40275 | 40275 | 22937 |
| Oryctes sp. | DBB3 | Embu | 85075 | 73948 | 73138 | 70104 | 70104 | 39865 |
| Oryctes sp. | DBA3 | Murang’a | 94882 | 82324 | 81008 | 75681 | 75681 | 39085 |
| Oryctes sp. | DBR1 | Nairobi | 58090 | 55578 | 52987 | 45774 | 45774 | 29618 |
| *C. aurata* | FBB4 | Embu | 38886 | 37036 | 36415 | 35106 | 35106 | 21914 |
| *C. aurata* | FBA4 | Murang’a | 51924 | 49275 | 47391 | 43180 | 43180 | 22393 |
| *C. aurata* | FBB5 | Embu | 60226 | 57386 | 55956 | 52788 | 52788 | 24424 |
| *C. aurata* | FBA5 | Murang’a | 94058 | 81495 | 79252 | 73815 | 73815 | 39895 |
| *C. aurata* | FBR2 | Nairobi | 88289 | 77450 | 73617 | 61765 | 61765 | 24576 |

**S14 Table**: Overview of the beetle larvae specimens examined in this study. Table includes species identity, collection locality and the number of fungal sequence reads for individual samples after filtering, denoising, merging, tabling as ASVs and removal of chimeras.

| **Species** | **Sample ID** | **Collection locality** | **Input** | **Filtered** | **Denoised** | **Merged** | **Tabled** |
| --- | --- | --- | --- | --- | --- | --- | --- |
| Oryctes sp. | DBA1 | Murang’a | 91365 | 85069 | 85000 | 83315 | 83315 |
| Oryctes sp. | DBB1 | Embu | 46141 | 42313 | 42267 | 42003 | 42003 |
| Oryctes sp. | DBR1 | Nairobi | 56018 | 53496 | 53457 | 52774 | 52774 |
| *C. aurata* | FBB4 | Embu | 70512 | 67221 | 67000 | 65727 | 65727 |
| *C. aurata* | FBA4 | Murang’a | 60716 | 57434 | 57351 | 56542 | 56542 |
| *C. aurata* | FBR2 | Nairobi | 120147 | 116829 | 116753 | 112505 | 112505 |
