# Supplementary figures and images for: Elucidating the functions of gut microbiota from two edible dung beetle species: Implications for waste management and food industry
Source: PLoS One. 2025 Jun 25;20(6):e0325756. doi: 10.1371/journal.pone.0325756 (PMC12193771; doi:10.1371/journal.pone.0325756)

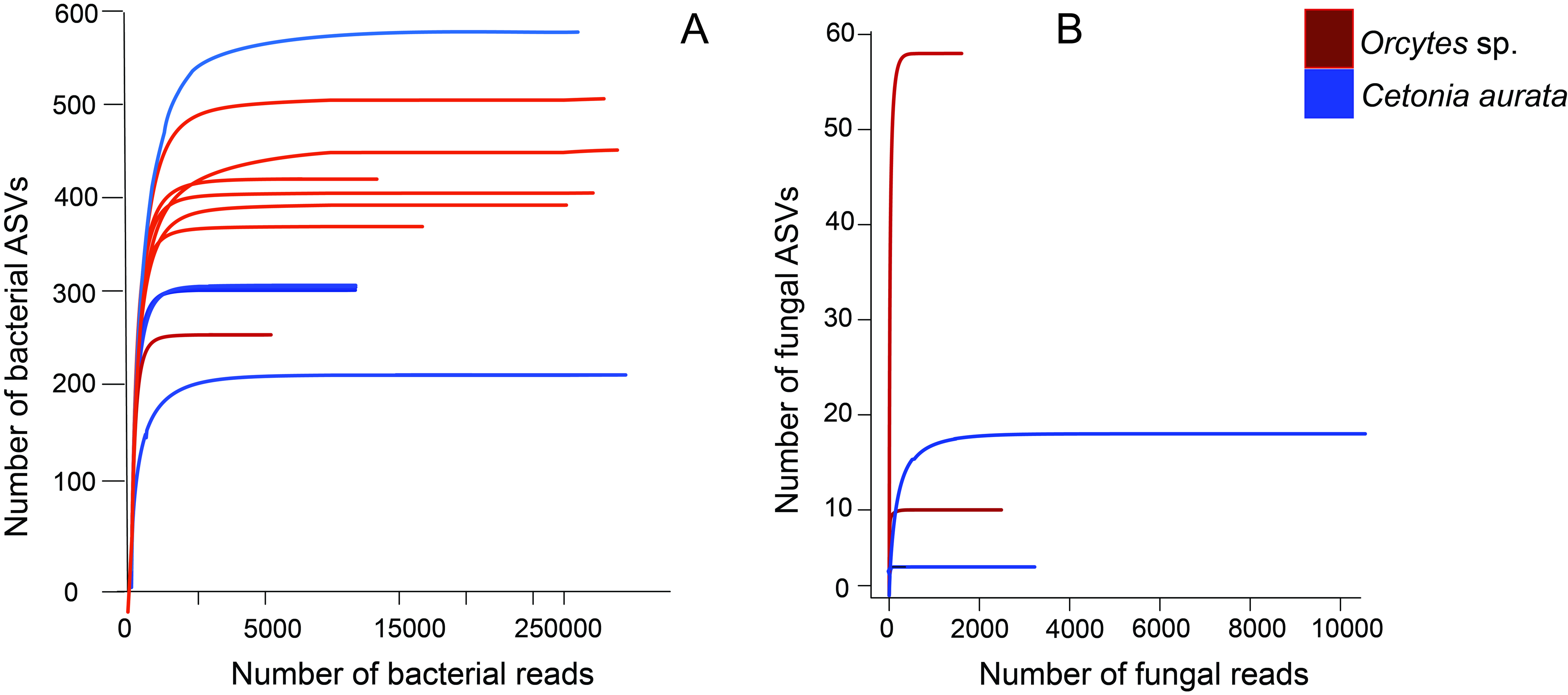

Supplement: S1 File — Supporting information has been provided (Tables S1- S11 and Figures S1-S3). (ZIP) [file pone.0325756.s001.zip › Suppl/S1_Fig.tif]

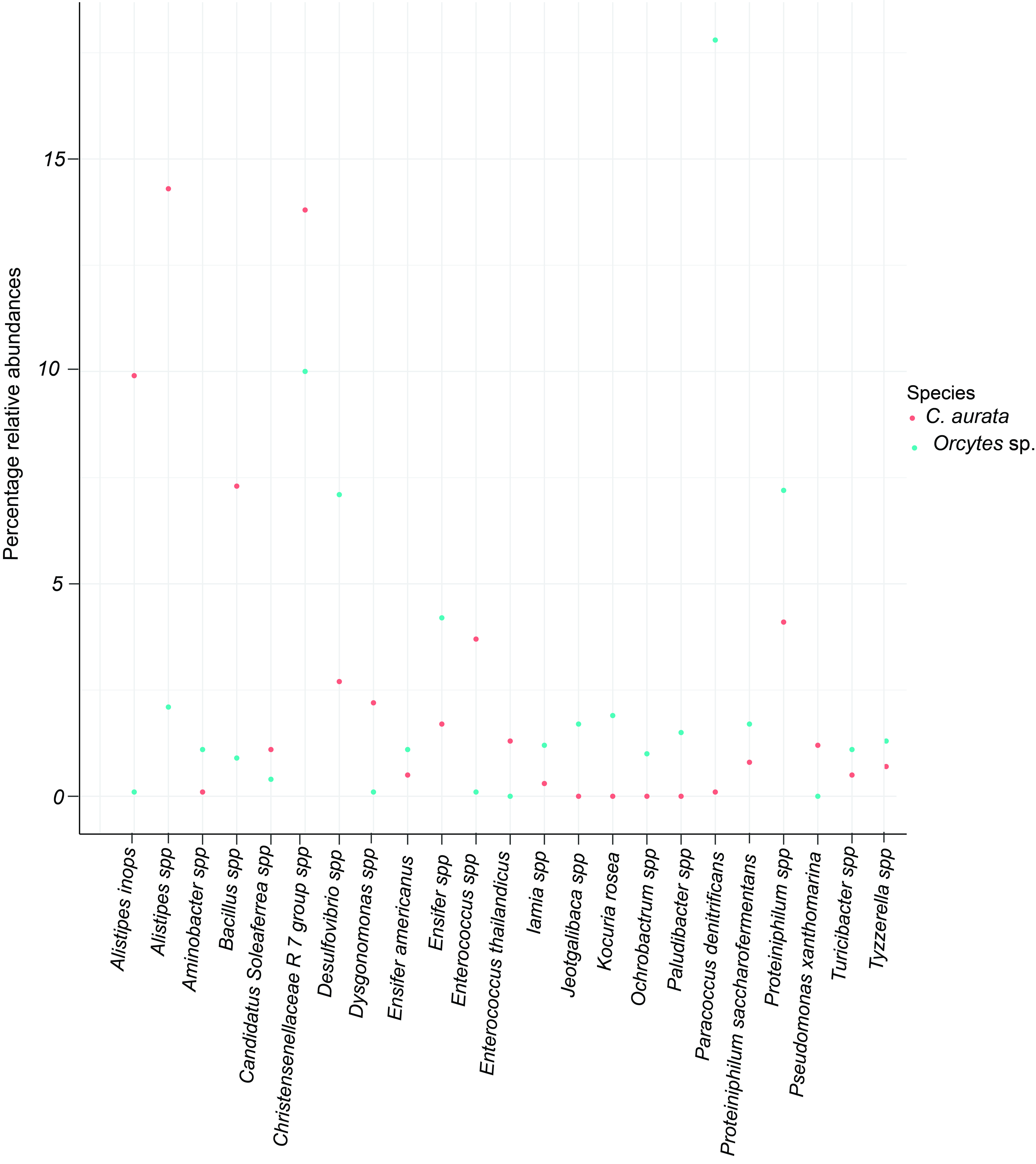

Supplement: S1 File — Supporting information has been provided (Tables S1- S11 and Figures S1-S3). (ZIP) [file pone.0325756.s001.zip › Suppl/S2_Fig.tif]

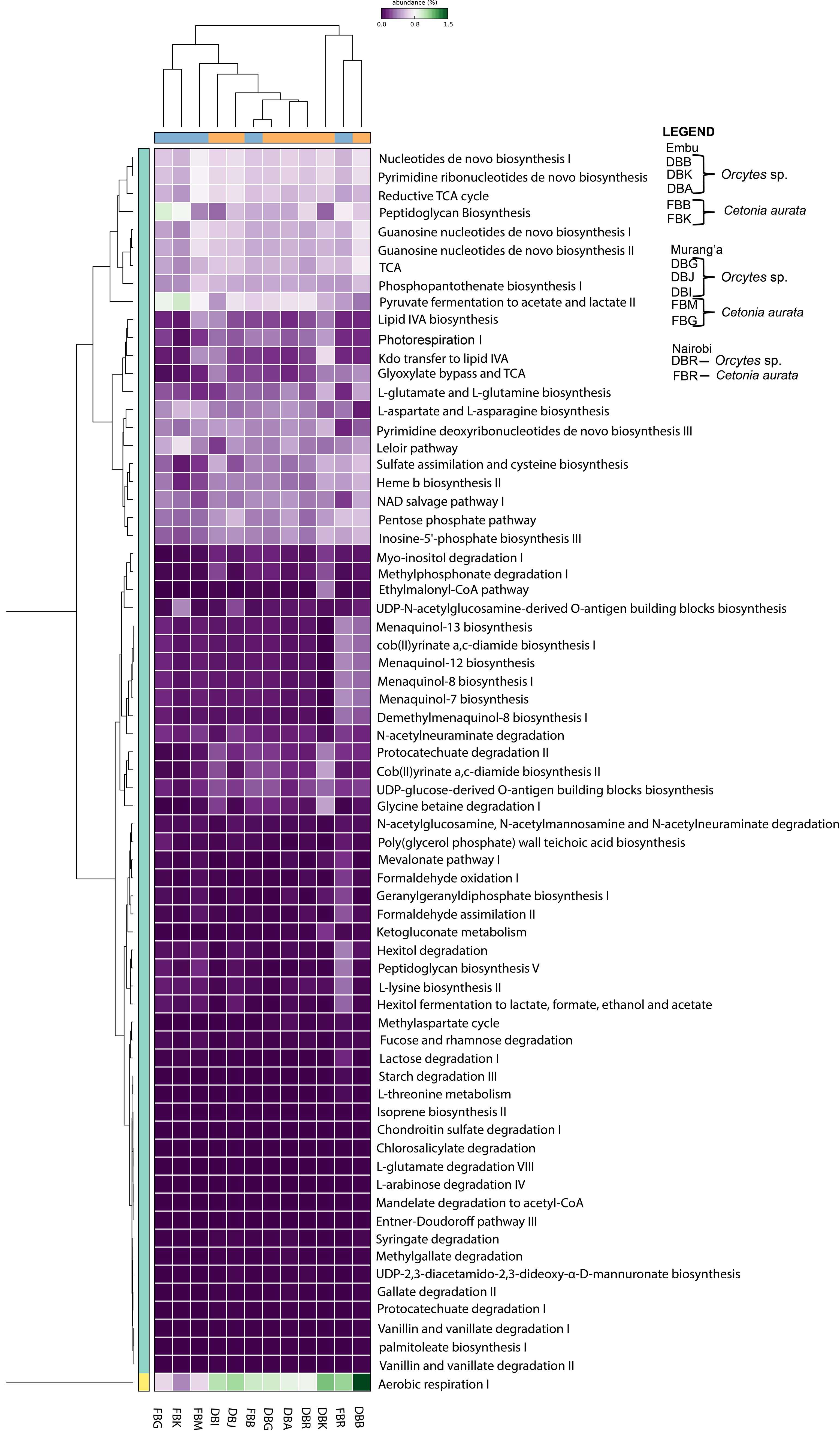

Supplement: S1 File — Supporting information has been provided (Tables S1- S11 and Figures S1-S3). (ZIP) [file pone.0325756.s001.zip › Suppl/S3_Fig.tif]
